# Real‑world survival outcome comparing abiraterone acetate plus prednisone and enzalutamide for nonmetastatic castration‐resistant prostate cancer

**DOI:** 10.1002/cam4.6536

**Published:** 2023-09-14

**Authors:** Takuya Tsujino, Satoshi Tokushige, Kazumasa Komura, Wataru Fukuokaya, Takahiro Adachi, Yosuke Hirasawa, Takeshi Hashimoto, Atsuhiko Yoshizawa, Masanobu Saruta, Takaya Ohno, Keita Nakamori, Ryoichi Maenosono, Kazuki Nishimura, Shogo Yamazaki, Taizo Uchimoto, Takafumi Yanagisawa, Keiichiro Mori, Fumihiko Urabe, Shunsuke Tsuzuki, Kosuke Iwatani, Shutaro Yamamoto, Kiyoshi Takahara, Teruo Inamoto, Takahiro Kimura, Yoshio Ohno, Ryoichi Shiroki, Haruhito Azuma

**Affiliations:** ^1^ Department of Urology Osaka Medical and Pharmaceutical University Takatsuki Japan; ^2^ Department of Urology The Jikei University School of Medicine Minato‐ku Japan; ^3^ Department of Urology Tokyo Medical University Shinjuku‐ku Japan; ^4^ Department of Urology Fujita‐Health University School of Medicine Toyoake Japan

**Keywords:** abiraterone acetate, androgen receptor signaling inhibitor, castration‐resistant prostate cancer, enzalutamide, nonmetastatic castration‐resistant prostate cancer

## Abstract

**Background:**

There is little evidence of abiraterone acetate (AA) plus prednisone for patients with non‐metastatic castration‐resistant prostate cancer (nmCRPC). In this study, we conducted a comparative analysis of real‐world survival outcomes between AA plus prednisone and enzalutamide (Enz) in patients with nmCRPC, utilizing our consortium dataset.

**Materials and Methods:**

The clinical records of 133 nmCRPC patients treated with first‐line Enz or AA plus prednisone were analyzed. The primary endpoints of the study were overall survival (OS) and cancer‐specific survival (CSS). Cumulative incidence function (CIF) using Fine and Gray models was also utilized to assess non‐cancer‐caused death considering the competing risk of cancer‐caused death.

**Results:**

During a median follow‐up of 36 months, 34 patients (25.6%) had deceased, with a median OS of 99 months in the entire cohort. There were no significant differences in comorbidities between the Enz and AA groups. Time to PSA progression (TTPP: HR 0.81, 95% CI 0.51–1.30, P = 0.375) and CSS (HR 1.32, 95% CI 0.55–3.44, P = 0.5141) were comparable between the two groups. However, intriguingly, there was a trend towards shorter OS in patients treated with AA plus prednisone compared to Enz (HR 0.57, 95% CI 0.29–1.12, P = 0.0978, median of 99 and 69 months in Enz and AA groups, respectively). CIF analysis revealed that nmCRPC patients treated with AA plus prednisone were more likely to result in non‐cancer‐caused death than those treated with Enz (HR 5.22, 95% CI 1.88–14.50, P = 0.0014).

**Conclusions:**

Our real‐world survival analysis suggests that while AA plus prednisone may demonstrate comparable treatment efficacy to Enz in the context of nmCRPC, there may be an increased risk of non‐cancer‐caused death. Physicians should take into consideration this information when making treatment decisions for patients with nmCRPC.

## Graphical abstract

In the daily clinic, ARSIs, including AA plus prednisone, are offered to a different patient population from randomized controlled trials as the elder and more comorbidities exist. Whether AA plus prednisone for nmCRPC elicits benefits on OS has not been fully proven. We report the real‐world survival outcomes comparing AA plus prednisone and Enz for nmCRPC and revealed that non‐cancer‐caused death might increase for patients treated with AA plus prednisone compared to Enz.

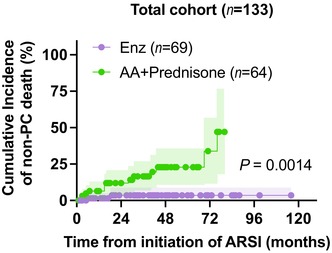

## 1 INTRODUCTION

Prostate cancer (PC) constitutes the second most prevalent cancer diagnosis in males and the sixth principal cause of cancer‐associated mortality worldwide.^1^ Androgens serve a pivotal function in fostering PC cell growth, rendering androgen deprivation therapy (ADT) the foremost treatment modality for men with PC, in addition to surgical and radiation therapies.^2^, ^3^ Although initially efficacious, patients receiving ADT for PC inevitably exhibit resistance, culminating in castration‐resistant prostate cancer (CRPC), frequently without discernible metastases on traditional imaging.^4^, ^5^ In recent years, the development of drugs aimed at extending the lives of CRPC patients has expanded to include second‐generation androgen signaling receptor inhibitors (ARSIs). Three phase 3 randomized clinical trials (RCTs) demonstrated the effectiveness of ARSIs, such as SPARTAN (apalutamide [Apa]), PROSPER (enzalutamide [Enz]), and ARAMIS (darolutamide [Dar]), in treating patients with nonmetastatic CRPC (nmCRPC).^6^, ^7^, ^8^ All three agents, which are potent androgen receptor inhibitors, have demonstrated significantly prolonged duration to clinical progressions, such as metastasis emergence, the elevation of PSA level, and symptom progression.^7^, ^9^ Another type of ARSI, abiraterone acetate (AA), is a selective CYP17 inhibitor that biosynthetically suppresses androgen levels.^10^ It is utilized in conjunction with prednisone to mitigate the adverse effects of excessive mineralocorticoid activity.^11^

AA plus prednisone and Enz are now used worldwide for patients with metastatic CRPC (mCRPC).^12^, ^13^ To our knowledge, only one trial, IMAAGEN (NCT01314118: a single arm phase 2 study), had been conducted on AA plus prednisone for nmCRPC patients, demonstrating a comparable effect to other ARSIs.^14^ However, the IMAAGEN trial did not include OS as their outcome measure and concluded that survival outcomes must be validated by subsequent studies. The latest network meta‐analysis of ARSIs for nmCRPC, including AA plus prednisone for nmCRPC, indicated that AA plus prednisone offers comparable metastasis‐free survival benefit but also shows the highest odds of serious adverse events (Hazard ratio [HR] 1.94, 95% confidence interval [CI] 1.17–3.22) compared to other patent‐protected ARSIs.^15^ Furthermore, in the daily clinic, ARSIs are generally offered to a different patient population from RCTs as the elder and more comorbidities exist. Thus, whether AA plus prednisone for nmCRPC elicits benefits on overall survival is still unknown. In Japan, AA plus prednisone has been offered to nmCRPC patients with the national healthcare insurance system since 2014. Herein, we report the real‐world survival outcomes comparing AA plus prednisone and Enz for nmCRPC utilizing our multi‐institutional dataset.

## 2 MATERIALS AND METHODS

This retrospective study was conducted using multi‐institutional cohorts, including Osaka Medical and Pharmaceutical University (Osaka, Japan), Tokyo Medical University, the Jikei University School of Medicine (Tokyo, Japan), and Fujita‐Health University School of Medicine (Aichi, Japan). The research design received approval from the institutional review board of Osaka Medical and Pharmaceutical University (IRB approval number: RIN‐750‐2571, approval date: January 24, 2020) and was conducted in accordance with the World Medical Association Declaration of Helsinki. The clinical records were collected retrospectively. Inclusion criteria were patients who underwent first‐line Enz or AA plus prednisone treatment for nmCRPC (including regional lymph node metastases: N1 cases). Clinical records of 133 consecutive patients diagnosed with nmCRPC were collected. Of 133 nmCRPC patients, patients were treated with first‐line Enz (*n* = 69, 51.9%) and AA plus prednisone (*n* = 64, 48.1%). Clinical variables in the present study involve age (in years), time to castration resistance (TTCR; in months), time to PSA progression (TTPP; in months), baseline PSA level (in ng/mL) at CRPC diagnosis, PSA doubling time (PSADT; in months), eastern cooperative oncology group performance status (ECOG‐PS; 0/≥1), loco‐regional disease (N0/N1), local treatment (−/+), and duration of treatment (in months). At each of the participating institutions, PSA values were quantified using automated chemiluminescent immunoassays (CLIA) employing the ARCHITECT Total PSA Calibrators 7K70‐01, manufactured by ABBOTT Laboratories.

PSA progression was determined based on the PCWG2 guidelines, which state a serum testosterone level of <50 ng/dL combined with either PSA progression (a rise of 25% and an absolute increment of 2 ng/mL or higher above the PSA nadir) or radiographic progression.^16^ The clinical stage was assessed utilizing magnetic resonance imaging (MRI), computed tomography (CT), and bone scintigraphy, ensuring the absence of discernible metastasis at the time of CRPC diagnosis. Lymph node metastasis was characterized as >15 mm in accordance with the RECIST guidelines (version 1.1).^17^ All other clinical variables, encompassing age, TTCR, PSA value, PSADT, and ECOG‐PS, were recorded upon CRPC diagnosis.

Enz and AA plus prednisone were administered at a standard dose.^7^, ^18^ Dose adjustments and treatment intervals were individualized based on the patient's overall condition and side effects, as determined by the physician. Follow‐up CT scans were performed quarterly after the CRPC diagnosis to detect potential signs of disease progression. Additional imaging modalities, such as MRI, bone scintigraphy, and PET/CT scans, were utilized as needed to confirm the diagnosis of disease progression. The primary outcomes of the investigation included OS and CSS. In this study, OS was assessed from the initiation of first‐line ARSI therapy for nmCRPC patients until the final follow‐up or death. The determination of death, whether cancer‐caused or non‐cancer‐caused, was made based on information from the death certificate as determined by the attending physician. Specifically, patients who died without disease progression, such as the development of metastasis, were classified as non‐cancer‐caused deaths. Serum PSA concentrations were evaluated at a minimum of once every 3 months.

Fisher's exact test was executed to evaluate the relationship between each variable using a contingency table. For continuous variables, differences were assessed through Student's *t*‐test or the Wilcoxon test. Survival rates were estimated using Kaplan–Meier methods. A log‐rank test was carried out to identify clinical disparities between categorized cohorts. HRs and corresponding 95% CIs were derived from Cox proportional‐hazard regression models and depicted as a forest plot. Fine and Gray models were constructed to analyze the cumulative incidence function (CIF) of non‐PC‐related fatalities, accounting for the competing risk of PC‐related deaths. All statistical tests were two‐tailed, with *p* < 0.05 deemed to indicate statistical significance. Analyses were conducted utilizing GraphPad Prism software (GraphPad Software, La Jolla, CA, USA) and R Statistical Software (v4.1.2, R Core Team 2021).

## 3 RESULTS

### 3.1 Follow‐up and clinical characteristics

The median follow‐up time from the initiation of ARSIs for all patients was 36 months. The clinical characteristics of all 133 nmCRPC patients are shown in Table 1. There were 69 (51.9%) and 64 patients (48.1%) who were treated with Enz (median follow‐up: 35 months) or AA plus prednisone (median follow‐up: 36.5 months) as the first‐line treatment for nmCRPC, respectively. The median age at CRPC diagnosis was 79 years old. Local treatments had been performed in a total of 61 (45.9%) of 133 patients. The median treatment duration of first‐line Enz or AA plus prednisone was 14 and 11 months, respectively. During the follow‐up, 34 of 133 (25.6%) patients were deceased. All baseline backgrounds at the initiation of ARSIs were comparable between patients treated with Enz and AA groups. We also summarized the patient's comorbidities, including diabetes, old myocardial infarction, angina, congestive heart failure, hypertension, and cerebrovascular disease at initiating ARSIs (Enz and AA plus prednisone). As shown in Table 1, all comorbidities were observed to the same extent between Enz and AA groups.

**TABLE 1:** Patient characteristics at the initiation of ARSIs in 133 nmCRPC patients.

	Enzalutamide (*n* = 69)	Abiraterone plus prednisone (*n* = 64)	*p* value
Age, median years (range)	78 [54, 92]	80 [58, 101]	0.361
Smoking history			
Yes, *n*	33 (47.8)	28 (43.8)	0.855
No, *n*	30 (43.5)	28 (43.8)	
Unknown, *n*	6 (8.7)	8 (12.4)	
Time from initial diagnosis to CRPC, median months (range)	75.5 [3.0, 223.0]	61.0 [3.0, 488.0]	0.368
PSA at CRPC diagnosis, median ng/mL (range)	4.54 [0.06, 80.10]	3.47 [0.15, 21.64]	0.116
PSA doubling time, median months (range)	3.90 [0.70, 46.50]	3.65 [0.80, 44.30]	0.468
ECOG PS			
0, *n* (%)	44 (63.8)	39 (60.9)	0.858
≥1, *n* (%)	25 (36.2)	25 (39.1)	
Loco‐regional disease			
N0, *n* (%)	53 (76.8)	52 (81.2)	0.671
N1, *n* (%)	16 (23.2)	12 (18.8)	
Local treatment			
None, *n* (%)	37 (53.6)	35 (54.7)	0.540
Prostatectomy, *n* (%)	14 (20.3)	17 (26.6)	
Radiation therapy, *n* (%)	18 (26.1)	12 (18.8)	
Duration of treatment, median months (range)	14 [1, 86]	11 [1, 66]	
Diabetes			
Yes, *n* (%)	8 (11.6)	9 (14.11)	0.796
No, *n* (%)	61 (88.4)	55 (85.6)	
Old myocardial infarction			
Yes, *n* (%)	4 (5.8)	5 (7.8)	1
No, *n* (%)	65 (94.2)	59 (92.2)	
Angina			
Yes, *n* (%)	5 (7.2)	5 (7.8)	1
No, *n* (%)	64 (92.8)	59 (92.2)	
Congestive heart failure			
Yes, *n* (%)	7 (10.1)	6 (9.4)	1
No, *n* (%)	62 (89.9)	58 (90.6)	
Hypertension			
Yes, *n* (%)	34 (49.3)	35 (54.7)	0.603
No, *n* (%)	35 (50.7)	29 (45.3)	
Cerebrovascular disease			
Yes, *n* (%)	8 (11.6)	7 (10.9)	1
No, *n* (%)	61 (88.4)	57 (89.1)	

Abbreviations: ARSIs, androgen receptor signaling inhibitors; ECOG PS, eastern cooperative oncology group performance status; nmCRPC, nonmetastatic castration‐resistant prostate cancer; PSA, prostate specific antigen.

### 3.2 PSA response

**FIGURE 1:**
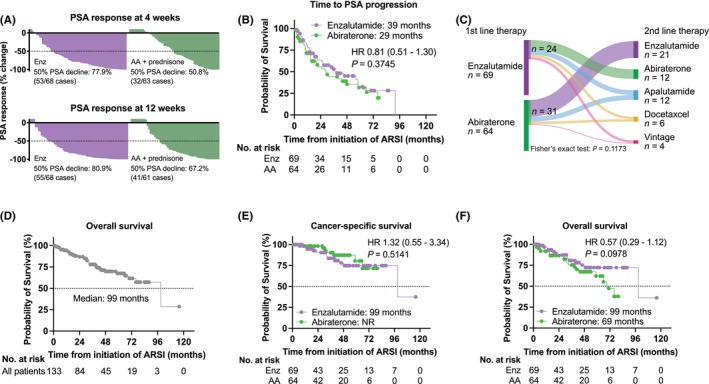
Survival outcome of nmCRPC patients treated with abiraterone plus prednisone and enzalutamide. (A) Confirmed PSA responses at 4 (upper panel) and 12 (lower panel) weeks after treatment initiation of enzalutamide (Enz) and abiraterone acetate (AA) plus prednisone. (B) Kaplan–Meier curves of time to PSA progression (TTPP) from treatment initiation. (C) Sankey diagram of the treatment sequences in nmCRPC patients treated with first‐line enzalutamide (*n* = 69) and abiraterone acetate plus prednisone (*n* = 64). (D) Kaplan–Meier curve of overall survival from treatment initiation of ARSIs in 133 nmCRPC patients (E, F) Kaplan–Meier curves of cancer‐specific survival (E) and overall survival (F) from treatment initiation of enzalutamide (*n* = 69) and abiraterone acetate plus prednisone (*n* = 64) in nmCRPC patients.

At the four‐week mark of treatment, 53 out of 68 patients in the Enz group (77.9%) and 32 out of 63 patients in the AA group (50.8%) showed a 50% decline in PSA levels, demonstrating a statistically significant difference (*p* = 0.0011, Figure 1A). At 12 weeks of the treatment, the 50% PSA decline was further confirmed in 55 (80.9%) of 68 patients and 41 (67.2%) of 61 patients in the Enz and AA groups, respectively (*p* = 0.0757, Figure 1A). No significant difference in TTPP was detected between the two groups, as the median TTPP reached 39 months for the Enz group and 29 months for the AA group (HR 0.81, 95% CI 0.51–1.30, *p* = 0.375, Figure 1B).

### 3.3 Subsequent therapy and survival outcomes

**FIGURE 2:**
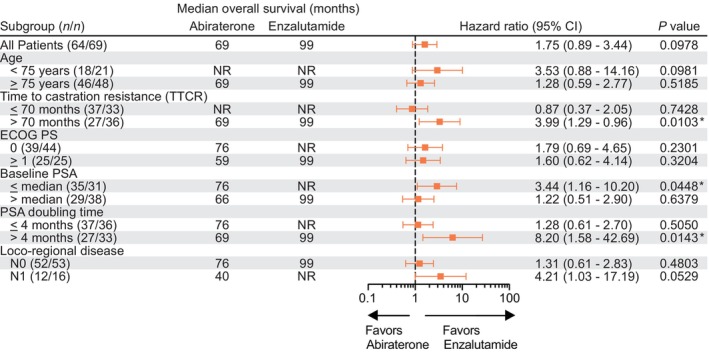
Forest plot subgroup analysis of overall survival by baseline patient characteristics. The analysis of all patients and all subgroup analyses were unstratified. ECOG PS, Eastern Cooperative Oncology Group performance status.

Fifty‐five (41.4%) of 133 patients underwent subsequent second‐line treatment during the follow‐up, with no significant difference between Enz (24 of 69: 34.8%) and AA (31 of 64: 48.4%) (*p* = 0.1173, Figure 1C). A median OS from the initiation of the first‐line ARSIs was 99 months (Figure 1D). Next, we investigated the impact of each treatment on survival. A comparable CSS was confirmed in Enz (median: 99 months) and AA (median: not reached) groups (HR 1.32, 95% CI 0.55–3.44, *p* = 0.5141) (Figure 1E). Inquiringly, there was a trend that patients treated with AA plus prednisone had shorter OS than those treated with Enz (Enz: median of 99 months, AA: median of 69 months, HR 0.57, 95% CI 0.29–1.12, *p* = 0.0978, Figure 1F). Furthermore, subgroup analysis of OS revealed that patients treated with Enz had longer OS than those treated with AA plus prednisone in the subgroups with a longer TTCR (*p* = 0.0103), lower baseline PSA value (*p* = 0.0448), and longer PSADT (*p* = 0.0143) (Figure 2).

## 4 CUMULATIVE INCIDENCE FUNCTION FOR NON‐PC CAUSED MORTALITY

**FIGURE 3:**
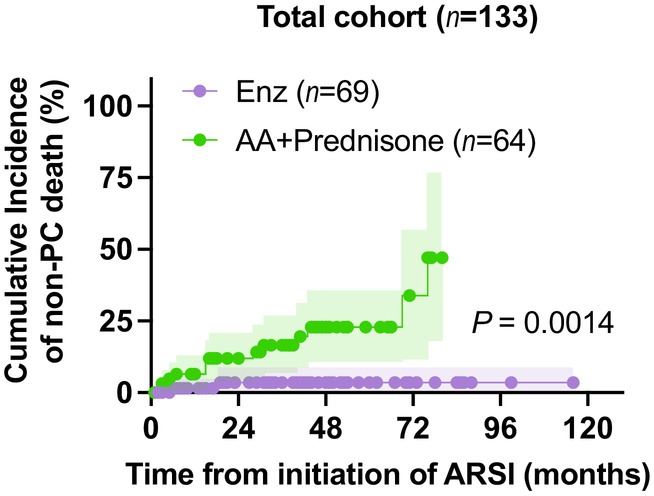
Non‐cancer mortality in nmCRPC patients treated with abiraterone plus prednisone and enzalutamide. Cumulative incidence plot of non‐cancer‐caused death from treatment initiation of enzalutamide (*n* = 69) and abiraterone acetate plus prednisone (*n* = 64) in nmCRPC patients; shaded areas represent 95% CIs.

Considering the competing risk of PC‐specific death, we adopted CIF analysis to examine whether the probability of non‐PC‐caused death could be different between Enz and AA groups. We found that nmCRPC patients treated with AA plus prednisone as the first‐line treatment were more likely to result in non‐PC‐caused death than those treated with Enz (HR 5.22, 95% CI 1.88–14.50, *p* = 0.0014, Figure 3). We further explored CIF in several subgroups that showed a significant difference in OS (Figure 2). Increased non‐PC caused death in nmCRPC patients treated with AA plus prednisone for the first‐line treatment was more likely to be observed in the subgroups of “TTCR > 70 months: *p* = 0.0027,” “baseline PSA ≤ median: *p* = 0.0097,” and “PSADT > 4 months: *p* = 0.0111” (Supplementary Figure S1). In total, non‐PC‐caused death occurred in 2 (2.9%) of 69 cases in Enz and 13 (20.3%) of 64 cases in AA groups. Table 2 summarizes the causes of non‐PC mortality. In the AA plus prednisone group, heart disease (four cases: 30.7%) and sepsis (four cases: 30.7%) were reported as their leading causes of death.

**TABLE 2:** Cause of non‐PC deaths in 133 nmCRPC patients treated with Enz or AA plus prednisone as their first‐line treatment.

	Total 15 cases	Enz (*n* = 69) 2 cases	AA plus prednisone (*n* = 64) 13 cases
Heart disease (i.e., heart failure, myocardial infarction, arrythmia), *n*	4	0	4
Sepsis, *n*	5	1	4
Asphyxia, *n*	2	1	1
Others (i.e., other cancer, unknown), *n*	4	0	4

Abbreviations: AA, abiraterone acetate; Enz, enzalutamide; nmCRPC, nonmetastatic castration‐resistant prostate cancer; PC, prostate cancer.

## 5 DISCUSSION

The present study reported the real‐world outcomes of Enz and AA plus prednisone administrated to nmCRPC patients. Our study revealed no significant difference between first‐line Enz and AA plus prednisone regarding PSA response and CSS from the initiation of ARSIs. In the case of patients with mCRPC, Tagawa et al. reported real‐world survival outcomes based on data from the Veterans Health Administration (VHA) database, showing that chemotherapy‐naive patients initiating therapy with Enz had a superior OS compared to those treated with AA plus prednisone.^19^ As for patients with nmCRPC, this study represents the first investigation of real‐world outcomes for AA plus prednisone versus Enz.

Cumulated evidence on the efficacy of Enz in treating nmCRPC has demonstrated a significant reduction in the risk of disease progression or death by 76% (HR 0.24, 95% CI 0.14–0.42, *p* < 0.0001) and a decrease in the risk of PSA progression by 82% (HR 0.18, 95% CI 0.10–0.34, *p* < 0.0001) when compared to bicalutamide in the phase 2 STRIVE trial.^20^ In addition, the phase 3 PROSPER trial showed that Enz combined with ADT resulted in a 27% lower risk of mortality compared to placebo plus ADT (HR 0.73, 95% CI 0.61–0.89, *p* = 0.001).^21^ Thus, the efficacy of Enz for nmCRPC patients has been well established. However, there is still little evidence of AA plus prednisone for patients with nmCRPC. The previous IMAAGEN phase 2 trial investigated a 50% reduction in PSA level as the primary endpoint in a single arm of AA plus prednisone treatment for high‐risk nmCRPC, demonstrating that 86.9% and 59.8% of patients achieved 50% and 90% declines of PSA at 6 months of treatment, respectively.^14^ The median duration of radiographic disease progression, as determined by sensitivity analyses, was 41.4 months, which is nearly on par with the outcomes of the other three trials—PROSPER, SPARTAN, and ARAMIS—for nmCRPC.^6^, ^7^, ^21^ Nevertheless, the IMAAGEN trial did not include OS as their outcome measure and concluded that survival outcomes must be validated by subsequent studies. The present study investigating the real‐world survival outcomes of nmCRPC patients treated with first‐line AA plus prednisone revealed that the treatment efficacy of AA plus prednisone was comparable to Enz. However, most strikingly, we found that there is a possibility that AA plus prednisone is associated with an increased risk of non‐PC‐caused death for nmCRPC patients, especially in patients who were likely to have long‐term medication, such as longer TTCR, lower baseline PSA, and longer PSADT (Figure 3). Indeed, those subgroup patients had a longer CSS than their counterparts (TTCR >70 vs. ≤70 months: *p* = 0.0433, baseline PSA ≤ median vs. > median: *p* = 0.0011, PSADT ≤4 vs. >4 months: *p* = 0.0067, data not shown). It should be noted that the IMAAGEN trial was designed for 5 mg prednisone with AA, whereas our real‐world data have complied with the guideline of the Japanese Urological Association (10 mg prednisone per day).

Cumulative incidence analyses showed that non‐PC deaths occurred in AA‐treated patients more frequently than in Enz‐treated patients (20.3% vs. 2.9%), without bias in patients' baseline comorbidities (Table 1). The leading causes of death were heart disease (30.7%) and sepsis (30.7%). For men with advanced PC, who already face a heightened risk of metabolic and cardiovascular incidents due to their older age and simultaneous utilization of chronic androgen deprivation,^22^, ^23^ adverse events stemming from AA plus prednisone or Enz therapy may significantly influence their overall well‐being and life quality. A recent study regarding adverse events with ARSIs for PC patients in high‐volume real‐world settings reported that men given AA plus prednisone (*n* = 2736) had a heightened likelihood of requiring emergency care or hospital admission in connection with diabetes, hypertension, or cardiovascular ailments compared to AA‐naive men (HR 1.77, 95% CI 1.53–2.05, *p* < 0.001).^24^ On the contrary, men who received Enz (*n* = 2466) were at relatively modest increased risk of those events compared to men who did not receive Enz (HR 1.22, 95% CI 1.01–1.48, *p* = 0.040). Given that the characteristics of patients treated with Enz and AA are nearly identical, the substantially different magnitude of risk suggests that treatment with AA plus prednisone may potentially lead to metabolic and cardiovascular undesirable occurrences. Another recent study, which compared the rate of hospitalization before and during treatment in mCRPC patients, identified a 22% increase in hospitalizations with AA compared to a 3% increase with Enz, despite being used in a younger population with less comorbid disease. Among the causes of hospitalization, AA was associated with a higher risk of infections: The analyses showed a 44% increase in urinary tract infections, a 114% increase in sepsis, and a 93% increase in pneumonia with AA compared to Enz when assessing the rate of infections during treatment relative to 1 year prior (*p* < 0.0001).^25^ As typified by sepsis, glucocorticoids, co‐administered with AA, stand as another significant cause of these adverse events and are known contributors to sepsis.

The population‐based investigation revealed that, in comparison with individuals with the same underlying condition but not subjected to glucocorticoids, the adjusted HRs for infections exhibiting a notably increased risk in the glucocorticoid‐exposed group varied from 2.01 (95% CI 1.83–2.19, *p* < 0.001) for skin cellulitis to 5.84 (95% CI 5.61–6.08, *p* < 0.001) for lower respiratory tract infection.^26^ Notably, the presence of cancer as an underlying disease was strongly associated with an elevated risk of septicemia (HR 11.15, 95% CI 5.78–21.53, *p* < 0.001). In a separate administrative analysis, another group reported that in men with CPRC, cumulative corticosteroid exposure was associated with a significantly higher risk of developing an infection (high exposure vs. no exposure, adjusted HR 2.55, 95% CI 2.27–2.85, *p* < 0.001).^27^ In the present study, the non‐PC mortality rate was relatively higher than in the previous RCTs. This might be attributed to the differences in patient population, particularly elderly in the real‐world setting, and variations in safety monitoring between clinical trials and real‐world settings.^28^, ^29^ Together, our findings support the efficacy of AA as suggested by IMAAGEN trial, but also highlights possible adverse events.

The retrospective design and relatively small sample size of this study may introduce selection bias, which are important limitations. For example, in low‐risk or younger patients, the use of AA may result in a different adverse event profile. Considering the significant number of censoring cases before the median survival in the present cohort, the immaturity of the present cohort should be counted as a limitation. Thus, comprehensive and prospective investigations are necessary to validate our preliminary findings. Thus, comprehensive and prospective investigations are necessary to validate our preliminary findings.

## 6 CONCLUSION

The results in the present study highlight important outcomes regarding AA plus prednisone for nmCRPC patients. Physicians should be encouraged to incorporate this knowledge into their patient selection and treatment decision‐making for nmCRPC patients, and more careful follow‐up after initiation of AA plus prednisone treatment is critical for optimal therapeutic outcomes.

## AUTHOR CONTRIBUTIONS

**Takuya Tsujino:** Conceptualization (equal); data curation (equal); formal analysis (equal); investigation (equal); methodology (equal); project administration (equal); software (equal); visualization (equal); writing – original draft (equal). **Satoshi Tokushige:** Conceptualization (equal); formal analysis (equal); writing – original draft (equal). **Kazumasa Komura:** Conceptualization (equal); formal analysis (equal); funding acquisition (lead); investigation (equal); project administration (equal); supervision (equal); visualization (equal); writing – original draft (equal). **Wataru Fukuokaya:** Data curation (equal); formal analysis (equal); software (equal). **Takahiro Adachi:** Data curation (equal). **Yosuke Hirasawa:** Data curation (equal). **Takeshi Hashimoto:** Data curation (equal); investigation (equal); project administration (equal). **Atsuhiko Yoshizawa:** Data curation (equal). **Masanobu Saruta:** Data curation (equal). **Takaya Ohno:** Data curation (equal). **Keita Nakamori:** Data curation (equal). **Ryoichi Maenosono:** Data curation (equal). **Kazuki Nishimura:** Data curation (equal). **Shogo Yamazaki:** Data curation (equal); investigation (equal). **Taizo Uchimoto:** Data curation (equal); formal analysis (equal). **Takafumi Yanagisawa:** Data curation (equal). **Keiichiro Mori:** Data curation (equal). **Fumihiko Urabe:** Data curation (equal). **Shunsuke Tsuduki:** Data curation (equal). **Kosuke Iwatani:** Data curation (equal). **Shutaro Yamamoto:** Data curation (equal). **Kiyoshi Takahara:** Conceptualization (equal); supervision (equal). **Teruo Inamoto:** Supervision (equal). **Takahiro Kimura:** Conceptualization (equal); investigation (equal); project administration (equal); supervision (equal). **Yoshio Ohno:** Supervision (equal). **Ryoichi Shiroki:** Supervision (equal). **Haruhito Azuma:** Conceptualization (equal); project administration (equal); supervision (equal).

## FUNDING INFORMATION

This research was partially funded by the Grant‐in‐Aid No. 21H03070 (Japan Society for the Promotion of Science: JSPS), the SGH Research Foundation, and the Suzuken Memorial Foundation.

## CONFLICT OF INTEREST STATEMENT

The authors have no conflict of interest.

## ETHICS STATEMENT

Approval of the research protocol by an Institutional Reviewer Board: the research methodology was sanctioned by the Institutional Review Board of Osaka Medical and Pharmaceutical University (Approval number: RIN‐750‐2571, Approval date: January 24, 2020) and executed in accordance with the principles outlined in the World Medical Association Declaration of Helsinki, with the participants' informed consent.

Informed Consent: Written informed consent was obtained from the patients at enrolment in the study.

## Supporting information


Supplementary Figure 1.

## Data Availability

Data supporting the results of this research can be obtained from the corresponding author, subject to a justifiable request.
