# Supplementary material for: Real‑world survival outcome comparing abiraterone acetate plus prednisone and enzalutamide for nonmetastatic castration‐resistant prostate cancer
Source: Cancer Med. 2023 Sep 14;12(19):19414–22. doi: 10.1002/cam4.6536 (PMC10587977; doi:10.1002/cam4.6536)
Supplement: Supplementary file 1 — Supplementary Figure 1. [file CAM4-12-19414-s001.pdf]

Supplementary Fig.1

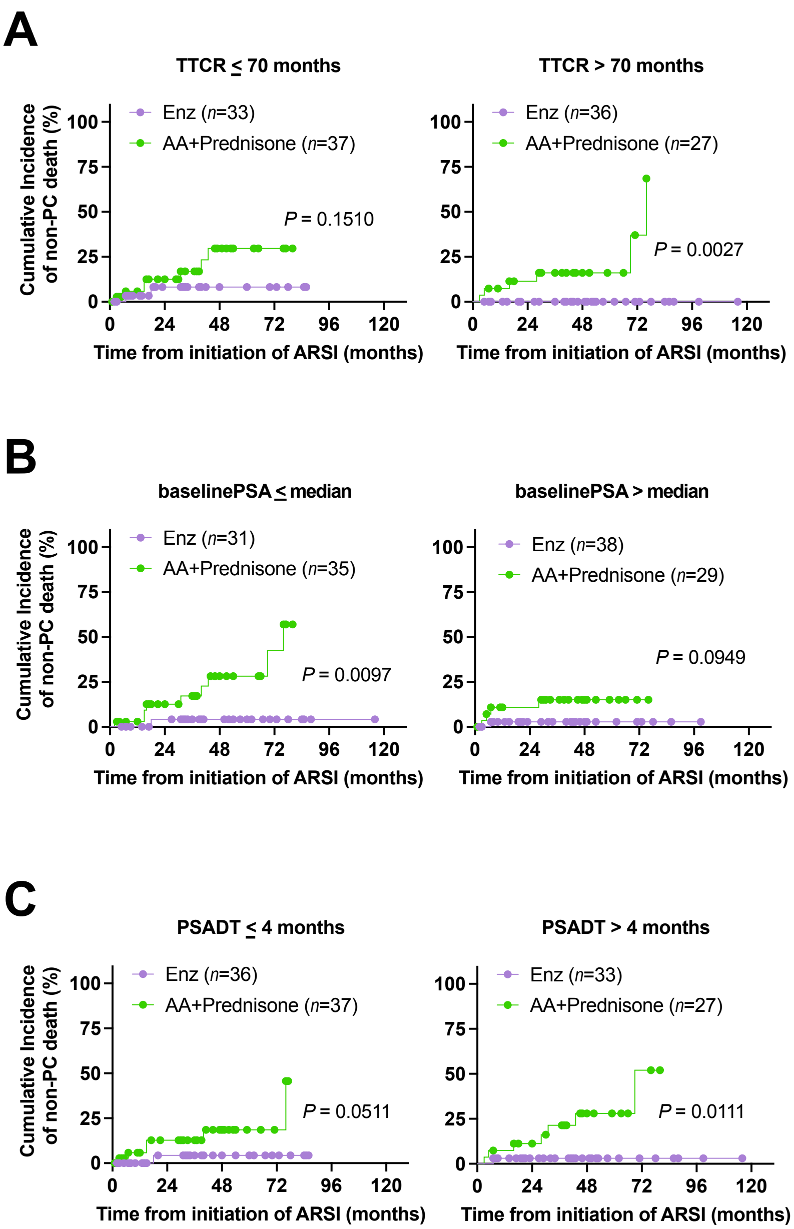

(A-C) Cumulative incidence plots of non-cancer-caused death from initiation of enzalutamide and abiraterone acetate plus prednisone in subgroups of time to castration resistance (B), baseline PSA value (C), and PSA doubling time (D).
